# How physical home workspace characteristics affect mental health: A systematic scoping review

**DOI:** 10.3233/WOR-220505

**Published:** 2023-10-19

**Authors:** Lisanne Bergefurt, Rianne Appel-Meulenbroek, Theo Arentze

**Affiliations:** Faculty of the Built Environment, Eindhoven University of Technology, Eindhoven, The Netherlands

**Keywords:** Workplace, teleworking, COVID-19 pandemic, psychological phenomena

## Abstract

**BACKGROUND:**

During the 1990s, voluntary teleworking became more grounded, because of expected advantages as increased productivity and comfort. However, COVID-19 obliged employees to work from home (WFH), even in unsuitable houses, which might have reduced their mental health. A holistic overview of methods and measures of the physical home-workspace characteristics and mental health is currently lacking. Insights in the potential influence of the physical home-workspace on mental health are also not yet holistically examined.

**OBJECTIVE:**

The aim of this study is to provide insights in previously studied relationships between the physical home-workspace and mental health and to identify measures for both using a systematic scoping review.

**METHODS:**

This study used the PRISMA method to systematically review existing literature.

**RESULTS:**

Most studies focussed on noise, acoustics, and privacy, in relation to productivity, concentration, and sleep quality. Only a few studies used objective measures for physical home-workspace characteristics.

**CONCLUSION:**

The list of relevant measures can be used by academics to examine relationships between the home-workspace and mental health further. Workplace managers can use it to help employees in optimizing their home-workspace.

## 1 Introduction

Telework can be defined as “a form of organising and/or performing work, using information technology in the context of an employment contract/relationship, where work, which could also be performed at the employer’s premises, is carried out away from those premises on a regular basis” [1]. This concept was introduced in the 1970s during the oil crisis, to reduce commuting and congestion in cities [2]. Telework became more grounded in the 1990s, due to the development of technology interventions at home, such as laptops and mobile phones. Traditionally, telework is believed to have several individual advantages. For instance, teleworkers might be more productive, because they experience fewer workspace distractions at home than at the office [3]. Furthermore, the home-workspace has been perceived as more comfortable, with better air quality, less noise, and more control over the temperature as the main benefits [4]. However, Ng [4] also indicated that telework might be a barrier for those who live in smaller-sized houses without a dedicated workroom.

During the COVID-19 pandemic, many employees were obliged to work from home (WFH) fulltime, even if their homes were not suitable for teleworking [5]. Xiao et al. [6] found that employees without a dedicated workroom or those who were dissatisfied with indoor environmental quality (IEQ) factors (e.g., noise, visual environment, air quality) reported new mental health issues during the pandemic, such as depression, stress, disengagement, mood, concentration, and sleep quality issues. Another COVID-19 study showed that satisfaction with daylight and artificial light, having a view outside, and greenery were also important for employees’ mental health, specifically for their concentration, mood, and well-being [7]. These findings show that, due to obligatory, fulltime WFH, it has become clear that home-workspace characteristics have become more important for employees’ mental health [5, 7]. Therefore, it is important to gain an overview of existing knowledge on this relationship for more evidence-based design of home workspaces for current hybrid working practices. So far, such overviews in relation to mental health are either only available for the office workplace [8] or are no systematic reviews [3, 4].

### 1.1 Objectives

This study aims to systematically review existing literature on the relationship between the physical characteristics of the home-workspace and mental health, to find out what is already known from contributions written between 1990 and now (2022). The novelty of this study, besides its focus on the home workplace, is that it uses a broad definition of mental health, introduced by Bergefurt et al. [8]. In addition, seven physical workspace characteristics that were described by Al Horr et al. [9] are used as search terms to obtain a holistic overview. Such an overview is valuable to set up future studies to advance this relationship. A second aim of this review is to identify which measures are used to study both the physical home-workspace as well as mental health. Insights from this study can be used by workplace managers to stimulate the optimization of the home-work environment for employees’ mental health. For academics, this study identifies important research gaps and relevant measures to be used in the home-workspace, and it provides holistic insights in potential workspace-health mechanisms.

## 2 Method

### 2.1 Search strategy

**Fig. 1:**
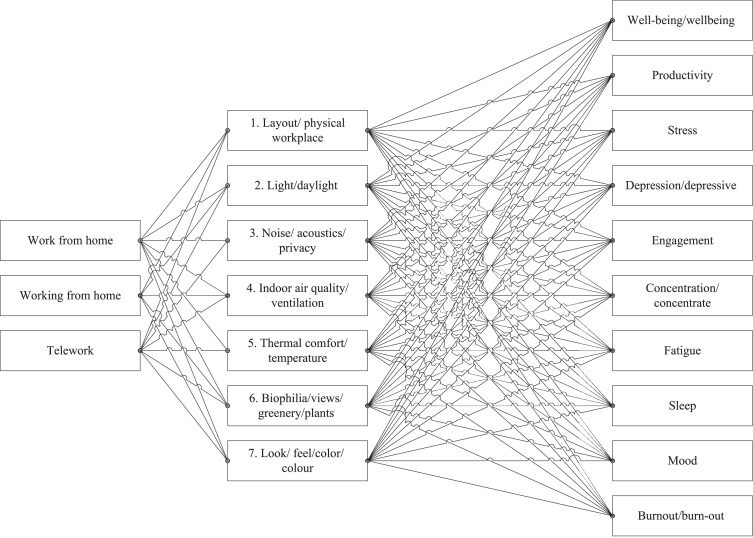
Search strategy.

For this systematic scoping review the PRISMA (Preferred reporting Items for Systematic Reviews and Meta-Analyses) guidelines were used to prepare the review protocol in advance [10]. The multidisciplinary citation database Scopus was used to generate the reviewed papers. After having searched for suitable papers in Scopus, both PubMed and Science Direct were used to search for additional contributions. The papers were selected based on a combination of terms as shown in Fig. 1. First, papers were selected that regarded working from home or teleworking. Then, seven of the eight physical workspace characteristics as introduced by Al Horr et al. [9] were used as search terms. They included location and amenities as the eighth workspace characteristic, but in the current review the focus is on the internal home-work environment. Therefore, the eighth characteristic has been disregarded. The seven physical workspace characteristics were combined with each of the mental health indicators, which were introduced by Bergefurt et al. [8]. For example, a search term looks like ‘work from home’ AND ‘noise’ AND ‘engagement’.

### 2.2 Study selection

**Fig. 2:**
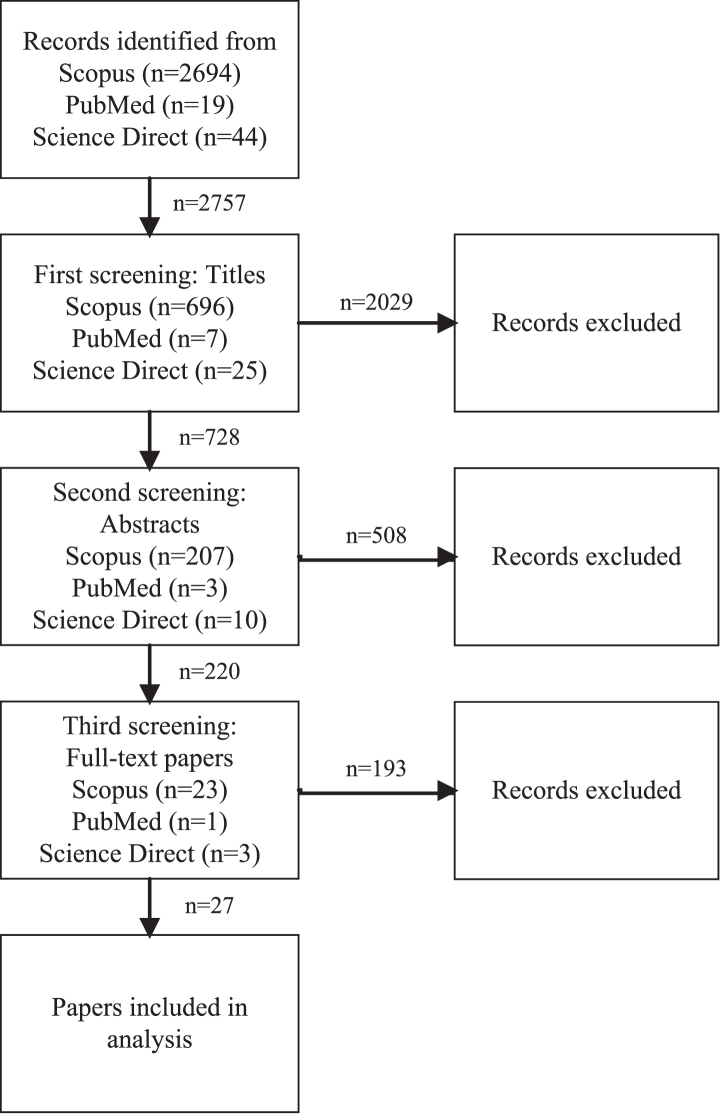
Screening process overview.

Figure 2 shows the screening and extraction process performed by the first author to select relevant papers, which was based on eligibility criteria that were set by all authors of this article. These criteria are that papers should (1) measure one of the physical home-workspace characteristics subjectively or objectively, (2) are conducted in the home-work environment or mimic such an environment, (3) measure at least one of the mental health outcomes subjectively or objectively, (4) are empirical studies with a longitudinal, prospective, or cross-sectional design, and (5) are available in English. Papers are deleted if they (1) do not report a physical home-workspace characteristic, (2) are not performed in the home-work environment or a mimic, (3) do not measure any mental health outcome, (4) are theoretical papers, reviews, or proceedings, or (5) are not fully available in English.

The screening process consisted of three phases, of which the first phase was title screening. In this phase, the total number of records identified from Scopus, PubMed, and Science Direct was reduced to 728. In the second phase, abstracts of the remaining 728 papers were read. Papers were deleted if they did not relate to the home-work environment, did not discuss any physical workspace characteristics, or were not related to mental health. In the third phase, the full text of the remaining 220 papers was read. In total, 193 papers were excluded, resulting in a final database of 27 papers. Papers were mainly deleted because the sample did not include (former) office workers, or because they did not describe a relationship between the physical workspace characteristics and mental health indicators. After the screening process, all authors of this study checked whether the final database met the eligibility criteria and whether important contributions were missing.

### 2.3 Quality assessment

The methodological quality of the papers was evaluated by the Mixed Methods Appraisal Tool (MMAT) [11]. MMAT can be used to appraise both qualitative, quantitative, and mixed method studies. For each paper, a quality score was calculated from 0 to 100%. For qualitative and quantitative studies, four criteria were defined, and the score was determined by the number of criteria that are met (i.e., one criterium met is 25%, all four criteria is 100%).

The criteria for qualitative studies include the relevance of the sources of data or the data analysis process to address the research questions, and if the research context or the researcher’s interaction with the participant influences findings. For quantitative studies, the criteria include whether participant recruitment minimizes selection bias and if the sample is representative of the population, if measures are tested on their validity and reliability (e.g., standard instruments), whether all key demographic information is summarized, and if the response rate is stated and acceptable.

For a mixed method approach, the score equalled the quality of the weakest component. The criteria include if the research design can address both qualitative and quantitative research questions, if both qualitative and quantitative data is collected and integrated, and whether the limitations (e.g., divergence of data) related to this integration are considered. The first author rated the methodological quality criteria individually, which were then discussed with the second and third author.

### 2.4 Data synthesis and analysis strategy

A data collection sheet was used to extract information from the included 27 studies. Information was extracted from each included paper on the following topics: 1.) general paper information (i.e., journal, publication year, sample size, research field, and continent); 2.) research approach, time horizon and methods; 3.) physical workspace characteristics and measures; 4.) mental health indicators and measures; 5.) direction of relationships between physical workspace characteristics and mental health indicators in the home-work environment.

This information was synthesized by counting the number of papers that were written in each year, published in a journal, or that used a specific research approach, time horizon and method. Then, four research fields were distinguished, and the included papers were divided over these fields. The papers were also summarized based on the continent in which they were written, and the average sample size was calculated. Furthermore, the number of papers that used specific measures of physical workspace characteristics and mental health indicators was summarized. The measures of physical workspace characteristics were divided in subjective and objective measures. Last, the direction of significant relationships between physical workspace characteristics and mental health indicators was identified.

## 3 Results

### 3.1 General paper information

**Fig. 3:**
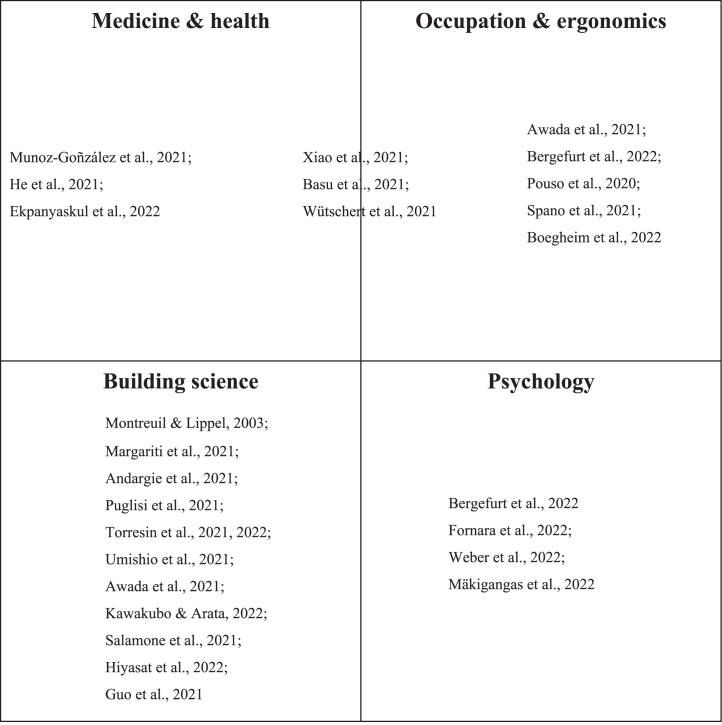
Thematic research fields.

Of the 27 papers, two were published in Applied Acoustics, two in Engineering Construction and Architectural Management, two in Journal of Environmental Psychology, and all others were published in different journals. Figure 3 shows the thematic research fields to which each of the included studies belong. Four research fields could be distinguished, namely medicine and health, occupation and ergonomics, building science, and psychology. Three studies combined the two research fields medicine and health and occupation and ergonomics. These were published in American College of Occupational and Environmental Medicine [6], Environmental Research Communications [12], and Industrial Health [13].

Fourteen papers were conducted in Europe, followed by six papers in Asia, four in North America, and two in multiple countries worldwide. Almost all studies were performed during the COVID-19 pandemic, except for one, namely Montreuil et al. [14]. The average number of respondents that participated in the included studies was 903, with 13 being the lowest [15] and 6080 being the highest [16].

### 3.2 Methodologies used

The Research Onion was used to categorize the research methodologies that were used in the included studies [17]. The onion distinguishes three layers, namely the time horizon layer, the methodological choice layer, and the research strategy layer. First, the time horizon layer can be divided in longitudinal, prospective, and cross-sectional approaches. In four studies, a longitudinal approach was used, meaning that one pre-test and at least two post-tests were performed [15, 18–20]. The remaining 23 studies used a cross-sectional approach, in which observations were measured at only one point in time.

The methodological choice layer can be divided in quantitative, qualitative, and mixed methods. In 23 studies, a quantitative method was used, meaning that data was analysed using statistical analysis. Three studies used a mixed method approach [15, 21, 22], in which quantitative data is complemented by qualitative data [21]. Only one study used a qualitative method, namely Montreuil and Lippel [14]. They used interviews and rating scales to understand why telework was adopted by an organization. This paper was the only study that was written before the COVID-19 pandemic.

The third layer, the research strategy, distinguishes experiments, interviews, and surveys. In 25 studies, a survey was used to obtain data. Four papers used interviews [14, 15, 21, 22], and three papers used an experiment [15, 18, 19]. Margariti et al. [15] asked participants to use a wearable band, which measured their physical activity and heart rate. They were also asked to rate their mood each day and to fill in a short daily diary. He et al. [18] used medical-grade actigraphy monitors to measure sleep and rest-activity cycles for five consecutive days. Third, Boegheim et al. [19] used wireless sensors that respondents should place on their desks at home to measure temperature-, relative humidity-, carbon dioxide-, sound pressure- and illuminance-levels.

The MMAT scores varied among included studies: two papers scored 100%, four scored 75%, eleven scored 50%, nine papers scored 25%, and only one study scored 0%. Table 1 shows an overview of the methodologies used and the MMAT scores. The study by Fornara et al. [23] was scored 100%, because the authors clearly explained the required sample size that was estimated by G*Power. They also used previously validated measurement scales and clearly described their sample. Mäkikangas et al. [20] also received a MMAT score of 100%, because they extensively described the statistical methods that they used to analyse the data. They also performed an attrition analysis to examine whether differences could be observed between the baseline at T1 and the final sample at T4. The study by Montreuil and Lippel [14] received a quality score of 0%, because their methodology was unclearly described and would therefore be hard to reproduce.

**Table 1:** Methodologies and quality assessment

Time horizon layer		Methodological choice layer			Research strategy layer			MMAT
	Longitudinal	Cross-sectional	Quantitative	Qualitative	Mixed method	Field experiment	Interview	Surveys	Quality score (%)
[5]		X	X					X	25
[6]		X	X					X	50
[7]		X	X					X	50
[12]		X	X					X	50
[13]		X	X					X	25
[14]		X		X			X		0
[15]	X				X	X	X		75
[16]		X	X					X	50
[18]	X		X			X		X	50
[19]	X		X			X		X	25
[20]	X		X					X	100
[21]		X			X		X	X	50
[22]		X			X		X	X	50
[23]		X	X					X	100
[24]		X	X					X	25
[25]		X	X					X	50
[26]		X	X					X	25
[27]		X	X					X	75
[28]		X	X					X	50
[29]		X	X					X	25
[30]		X	X					X	25
[31]		X	X					X	50
[32]		X	X					X	50
[33]		X	X					X	75
[34]		X	X					X	25
[35]		X	X					X	25
[36]		X	X					X	75

### 3.3 Frequency of relationships between physical home-workspace characteristics and mental health indicators

Table 2 shows the number of papers that studied the relationships between physical home-workspace characteristics and mental health indicators. In twenty studies, noise, acoustics, and privacy were studied in relation to mental health, mostly related to productivity (eleven times), stress (nine times), concentration (eight times), and sleep quality (eight times). The characteristic light and daylight was related most frequently to productivity (eight times) and sleep quality (seven times). Furthermore, thirteen studies considered thermal comfort and temperature, of which ten related it to productivity, and six to stress, concentration, sleep quality, or mood. Twelve studies considered indoor air quality and ventilation and related it mostly to productivity (eight times) and concentration (six times). In ten of the included papers, layout and design was studied in relation to mental health. It was related most frequently to productivity (four times), and concentration (three times). Biophilia and views was included in six studies, which were most often related to productivity (three times), depressive symptoms (three times), and mood (three times). Last, for look and feel, two studies investigated the relationship with mental health, both with productivity.

**Table 2:** Frequency of relationships studied between physical workspace and mental health

	Concentration	Sleep quality	Mood	Stress	Productivity	Depression	Well-being	Fatigue	Engagement	Burnout	Nr. of papers
Noise, acoustics, and privacy	8	8	7	9	11	5	6	5	5	3	20
Light and daylight	6	7	6	6	8	4	3	4	4	2	14
Thermal comfort and temperature	6	6	6	6	10	5	4	5	5	3	13
Indoor air quality and ventilation	6	5	5	5	8	4	3	4	4	2	12
Layout and design	3	1	2	2	4	1	2	2	2	2	10
Biophilia and views	2	2	3	2	3	3	1	1	1	1	6
Look and feel	1	1	1	1	2	1	1	1	1	1	2
**Nr. of papers**	10	10	9	10	12	7	6	5	6	4

### 3.4 Measures of physical workspace characteristics

#### 3.4.1 Noise, acoustics, and privacy

Table 3 shows the subjective and objective measures that were used for describing the physical workspace characteristics. All authors used subjective measures for noise, acoustics, and privacy. Several authors asked respondents to indicate their satisfaction with noise [5–7, 19, 24, 25] and privacy [7, 13, 23, 26]. In two papers, employees were asked to indicate how much noise annoyed them while WFH and which strategy they used to deal with these noise sources [27, 28], while another author asked teleworkers to indicate the annoyance caused by different noise sources before and after the COVID-19 pandemic [29]. Puglisi et al. [27] asked employees to rate their sensitivity and reaction to noise on a five-point scale. The Weinstein’s Noise Sensitivity Scale has also been used in two studied to assess teleworkers’ noise sensitivity [21, 22]. In another study, employees were asked to indicate why they chose a particular space to work from at home, which also included acoustic reasons (e.g., less noise or more privacy) [30].

**Table 3:** Objective and subjective measures of physical workspace characteristics

Objective measures		Subjective measures		Objective measures	Subjective measures
*Noise, acoustics, and privacy*				*Layout and design*
Sound pressure level	[19]	Satisfaction with noise	[5–7, 19, 24, 25, 32]	Presence dedicated workroom	[5]	Satisfaction with home	[23, 25]
		Satisfaction with privacy	[7, 13, 23, 30]	Size of workspace	[31]	Importance of dedicated work area	[14]
		Comfort of noise level	[15, 26, 31]	Size of home	[21, 22, 35]	Functionality of space as reason to WFH	[30]
		Influence noise on mental health, productivity	[27]	Private/ shared workspace	[31]	Functionality of space	[15, 20]
		Noise sensitivity	[27]	Crowdedness	[23]
		Types of noise	[21, 29]	Housing typology	[21, 22, 35]
		Noise annoyance	[14, 28]	Work area typology	[5]
		ISO/TS 12913-2	[21, 22]	Construction year home	[35]
		Weinstein’s Noise Sensitivity Scale	[21, 22]
		Acoustics as reason to WFH	[30]
*Light and daylight*				*Thermal comfort and temperature*
Light exposure levels	[18]	Satisfaction with natural light	[5–7, 23–25, 34]	Air temperature	[19, 32, 33]	Satisfaction with indoor air temperature	[5–7, 19, 24–26]
Illuminance levels	[19]	Satisfaction with electric light	[5–7, 24, 25]	Relative humidity	[32, 33]	Satisfaction with humidity	[6, 24]
		Satisfaction with glare	[5, 7, 24, 25]			Perception temperature	[28]
		Satisfaction with light environment	[32]			Thermal preference	[15, 31]
		Satisfaction with illuminance	[19]			Temperature as reason to WFH	[30]
		Perception of presence of light	[15]
		Comfort of light	[28]
		Artificial light as reason to WFH	[30]
*Biophilia and views*				*Indoor air quality and ventilation*
Access to outdoor space	[16, 34]	Satisfaction with greenery	[7]	CO2 concentration	[19, 32]	Satisfaction with air quality	[5–7, 19, 24–26, 28, 32]
Type of outdoor space	[34]	Satisfaction with views outside	[7, 25]	PM2.5 mass concentration	[32]	Satisfaction with ventilation	[7, 28]
Time spent in garden	[12]	Visual access to nature as reason to WFH	[30]			Evaluation of air quality (poor-fresh)	[15]
Composition of garden	[12]					Perception of air quality	[14, 26]
Type of view outside	[16]					Interference with air quality	[26]
Presence of plants	[34]					Air quality as reason to WFH	[30]
*Look and feel*
Wall colours	[31]	Wall colours as reason to WFH	[30]

Furthermore, several studies asked employees to indicate which noise sources they heard at home [21, 22, 27]. Torresin et al. [21, 22] adapted their question from the ISO/TS 12913-2 standard in both their studies to describe these noise sources (i.e., traffic noise, other noise from outside, natural sounds, human beings outside, other human beings present at home, neighbours, building services at home, building services of neighbours and common areas, and music/TV played by participants themselves). Another measure is the comfort level with background noise, building devices, and noise from outside the home [31]. Employees rated their comfort level on a five-point scale ranging from comfortable to uncomfortable [31], or from noisy to quiet [15]. In one study, respondents were also asked to rate the influence of noise on their mental health, well-being, and productivity [27]. Last, Boegheim et al. [19] objectively measured the sound pressure level by IEQ sensors that were placed on individuals’ home desks. Of all noise-related papers, most received a MMAT score of 50% (eight times), while only one received a score of 100% and one scored 0%. Overall, the quality of the noise-related studies is relatively high.

#### 3.4.2 Light and daylight

In several studies, satisfaction with the visual environment was calculated as the average score of satisfaction with natural light, electric light, and glare [5, 6, 24, 25, 28]. Fornara et al. [23] only included satisfaction with natural light in their study. Two authors measured satisfaction with the light environment in general [32, 33]. Umishio et al. [32] and Boegheim et al. [19] included the satisfaction with the illumination of the desk. Furthermore, Spano et al. [34] asked respondents to report their perception of the presence of sunlight on a three-point scale, ranging from not very bright to very bright, while Margariti et al. [15] asked them to rate the comfort of the light on a scale from dark to light. In another study, respondents were asked to indicate why they chose a particular space to work from at home, which also included access to daylight and sufficient artificiallight [30].

In only two studies objective measures of light and daylight were used [18]. In one study, the Troiano algorithm was described to calculate activity patterns, including activity levels, light (lux) exposure levels, and step counts. In another study, the illuminance level was measured by an IEQ sensor that was placed on employees’ desk at home [19]. Overall, most reviewed studies about light and daylight received a rather low MMAT score of 25% (five studies) or 50% (six studies), while the quality of only two studies was rated as 75% and of one studyas 100%.

#### 3.4.3 Thermal comfort and temperature

Of the thirteen studies about thermal comfort and temperature, five received a MMAT score of 25%, six of 50%, and two of 75%. The quality scores of these studies are relatively high. Satisfaction with the thermal environment was calculated as the average of the ratings for indoor air temperature and humidity [5, 6, 24]. In other studies, respondents were only asked to rate their satisfaction with the indoor temperature [7], or with the thermal environment in general [25, 33]. Furthermore, teleworkers’ evaluation of the indoor temperature was asked, ranging from cold to warm [15, 28, 31]. Salamone et al. [26] included both satisfaction with thermal comfort, employees’ preferences, perception, and interference with thermal comfort (i.e., ‘How does the thermal comfort of the environment interfere with your ability to work?’). Furthermore, Hiyasat et al. [30] asked respondents to indicate why they chose a particular space to work from, including the temperature at home. Last, in three studies, the indoor air temperature and relative humidity were objectively measured, using humidity loggers [32, 33] or wirelesssensors [19].

#### 3.4.4 Indoor air quality and ventilation

Most studies focussed on the subjective evaluation of the indoor air quality. In several studies, employees were asked to rate their satisfaction with the indoor air quality [5, 7, 14, 24, 25]. In one study, employees were also asked to rate their satisfaction with the ventilation in the home-work environment, on a five-point scale [7]. Furthermore, Margariti et al. [15] asked respondents to evaluate the air quality on a scale from poor to fresh. In another study, several subjective measures of the indoor air quality were used, including employees’ satisfaction, preferences, perception, and interference (i.e., ‘How does the indoor air quality interfere with your ability to work?’) [26]. Ekpanyaskul et al. [28] asked respondents to indicate environmental problems at home, including poor ventilation or air quality.

In one study humidity loggers were used to measure CO_2_ levels and PM_2.5_ mass concentration [32]. Boegheim et al. [19] used IEQ sensors to measure CO_2_ levels. The quality assessment of the papers about indoor air quality and ventilation shows that most studies received a somewhat low score of 0% (one study), 25% (four studies), 50% (five studies), or 75% (two studies).

#### 3.4.5 Layout and design

The studies that regarded layout and design had an average quality. Two studies received a MMAT score of 100% and one of 75%. Table 3 shows that most studies used objective measures for layout and design. In two studies, respondents were asked to indicate whether they had a dedicated room for work activities, a dedicated workspace with other uses, or whether they worked in a variety of spaces [5, 31]. Bergefurt et al. [31] also asked teleworkers whether they had a shared or private workspace, and to indicate the size of their workspace (i.e., small, medium, large). Other studies asked respondents to indicate the housing typology [21, 22, 35]. Torresin et al. [21, 22] specified the housing typology as detached single-family house, semi-detached or terraced house, or apartment block. They also asked teleworkers in which room they worked, including the bedroom, kitchen/living room, kitchen, or in a studio. Furthermore, Fornara et al. [23] measured the crowdedness as the number of occupants divided by the number of rooms in the home (i.e., people-per-room ratio). Other objective measures include the size and the construction year of the house [35].

Subjective measures include employees’ satisfaction with the space at home (i.e., in general, space/square footage, privacy, natural light) [23] or the satisfaction with the layout [25]. Another measure is the importance of setting up a workspace separate from family members [14, 15]. Margariti et al. [15] pointed to the conflicting priorities that teleworkers have between domestic and work life, especially when their homes are small. Hiyasat et al. [30] asked respondents to indicate why they chose a particular space to work from, including the functionality of the space, such as changing the furniture or layout of the space. Last, Mäkikangas et al. [20] included six items to measure the functionality of the home workspace, of which the following are most relevant: ‘I have an adequate space at home for remote working’, ‘I have necessary equipment at home for remote working’, ‘I can find enough peace at home for working’, and ‘I can maintain a healthy work-life balance when working from home’.

#### 3.4.6 Biophilia and views

Overall, the quality of the studies regarding biophilia and views was rated somewhat low, with no studies receiving a MMAT score of 75% or 100%. Several objective and subjective measures have been used. Some authors asked respondents to specify if they had access to an outdoor space at their home [12]. Pouso et al. [16] specified the outdoor space as none, a balcony or patio, or a shared or public area, while Spano et al. [34] divided it into having a terrace with green, a courtyard with green, a garden, having access to more than one type of outdoor space, or residing in the countryside or mountains. In another study, respondents were asked to indicate the time they spent on average in their home garden, and to describe the composition of their garden (i.e., ornamental plants, fruits, vegetables, flowering plants, herbs and spices, or medicinal plants) [12]. Bergefurt et al. [7] subjectively measured employees’ satisfaction with the greenery at their home. Furthermore, Hiyasat et al. [30] asked respondents to report the reason to choose to work from a particular space at home, including visual access to a natural or organic environment.

Next to measures related to biophilia, several authors used measures for views outside. One measure was the type of views that could be seen from the work area at home, specified as few views or urban views, mixed views, or natural views [16]. Some authors also asked employees to indicate how much views they had from their work area (i.e., no green view, little bit of view, some view but without trees, some view with trees, most of the view, all of the view) [34] or to rate the dominating vegetation in their view outside [21, 22]. Last, in two studies, the satisfaction with employees’ views outside was measured [7, 25].

#### 3.4.7 Look and feel

Only two studies included a measure of look and feel. In one study, employees were asked to indicate the wall colour of their workroom, in which they could choose between blue-green, red-warm, or white-neutral wall colours [31]. Hiyasat et al. [30] asked respondents to indicate why they chose a particular space to work from, including the colours in the room. These studies received a quality score of 25% or 50%.

### 3.4 Indicators of mental health

#### 3.4.1 Well-being

Table 4 shows the measures that were used for the mental health indicators. Two validated measures of well-being have been identified, namely the Health at Work Survey of WHO and the WHO-5 well-being index. In the Health At Work Survey, employees are asked to rate their well-being on a ten-point scale, ranging from low well-being to high well-being [7, 19, 31]. The WHO-5 well-being index consists of five questions related to the subjective psychological well-being of teleworkers [21, 22]. In another study, well-being was measured as quality of life, work happiness and satisfaction, work-life balance, and the absence of mental health problems while WFH [28].

**Table 4:** Measures of mental health indicators

**Concentration**		**Sleep quality**
Checklist individual strength	[7, 19, 31]	Sleep disturbance	[6, 24, 29, 34]
Perception of concentration	[14, 32]	Single-item scale sleep problems	[13]
Lack of concentration	[6, 24, 27, 34, 35]	Single-item sleep quality scale	[7, 19, 31]
		Athens Insomnia Scale	[32]
		Health at work survey of WHO	[7, 19, 31]
		Sleep diary	[18]
		Pittsburgh Sleep Quality Index	[18]
		Insomnia Severity Index	[18]
		Actigraphy	[18]
**Mood**		**Stress**
Perception of mood	[6, 24, 29, 34]	Mental stress	[6, 24, 28, 29]
UWIST mood adjective checklist	[7, 19, 31]	Depression, Anxiety, and Stress Scale	[12]
Plutchik’s wheel of emotions	[16]	Stress and worry	[7, 19, 31]
Circumplex model of affect	[15]	Patient and health questionnaire for depression and anxiety	[7, 19, 31]
		Perceived stress scale	[23]
		Noise as cause of stress	[27]
**Productivity**		**Depression**
Perceived productivity	[5, 25, 26, 30, 32, 36]	Depressive symptoms	[6, 24]
Health at work survey of WHO	[7, 19, 31]	Depression, Anxiety, and Stress Scale	[12]
Subjective assessment of workplace productivity	[33]	Four-item patient and health questionnaire for depression and anxiety	[7, 16, 19, 31]
Influence noise on productivity	[27]
Time management	[28]
Effective work hours	[28]
Work efficiency	[28]
Work output	[28]
Confidence in decision-making	[28]
Motivation to continue work	[28]
**Well-being**		**Fatigue**
Health at work survey of WHO	[7, 19, 31]	Perception of fatigue	[15, 24]
WHO-5 well-being index	[21, 22]	Checklist individual strength	[7, 19, 31]
Quality of life	[28]
Work happiness	[28]
Satisfaction	[28]
Work-life balance	[28]
Absence of mental health problems while WFH	[28]
**Engagement**		**Burnout**
Social engagement	[6, 24]	Oldenburg Burnout Inventory	[7, 19, 31]
Oldenburg Burnout Inventory	[7, 19, 31]	Exhaustion	[35]
Utrecht Work Engagement Scale	[20]

#### 3.4.2 Stress

In several studies, no existing scale has been used to measure stress [6, 27–29]. Puglisi et al. [27] asked respondents to fill in what their main feeling was related to noise and added stress as an answer option. In one study, the Depression, Anxiety, and Stress Scale (DASS-21) was used. This scale consists of seven items per construct. The stress-scale consists of items to measure teleworkers’ difficulties to relax, their nervousness, arousal, ease of being upset or agitated, irritability or over-reactivity, and their impatience [12]. Another measure of stress is the Perceived Stress Scale (PSS), which consists of ten items measured on a five-point Likert scale. It measures the degree to which situations in teleworkers’ life are perceived as stressful [23]. Furthermore, the Patient and Health Questionnaire for Depression and Anxiety (PHQ-4) can be used to measure stress. Two items, namely ‘feeling nervous, anxious or on edge’ and ‘not being able to stop or control worrying’ are related to stress. In three studies, these items are combined with two items of the Stress and Worry Scale (i.e., ‘feeling stressed’, ‘ruminating or agonising over things’) [7, 19, 31].

#### 3.4.3 Productivity

Several studies used a five-point Likert scale to measure employees’ perceived productivity [5, 26, 30, 32]. In another study, the influence of noise on productivity was measured with five items on a five-point scale [27]. Furthermore, Kawakubo et al. [33] developed a method to measure the subjective evaluation of productivity with four factors, namely productivity of information processing, productivity of knowledge processing, productivity of knowledge creation, and comprehensive productivity. They used the Subjective Assessment of Workplace Productivity (SAP) questionnaire, which can be used for the subjective evaluation of productivity. Guo et al. [25] included a more objective measure of productivity by examining respondents’ speed and accuracy of completing a task. In another study, six attributes of productivity were measured, namely time management, effective work hours, work efficiency, work output, confidence in decision-making, and motivation to continue work [28]. Furthermore, Weber et al. [36] developed a measure to observe changes in productivity during the pandemic while WFH compared to working at the office. Last, the Health at Work Survey of WHO was used in three studies to measure productivity [7, 19, 31]. This is a ten-point scale ranging from low productivity to high productivity.

#### 3.4.4 Concentration

In most studies, no existing scales were used to measure concentration. Respondents were mostly asked to indicate the extent to which they could concentrate on their work while WFH [6, 14, 24, 32, 34, 35]. In one study, respondents were asked to indicate the disorders they suffered from during the COVID-19 lockdown period [35]. Lack of concentration was one of the answer options. Furthermore, Puglisi et al. [27] asked teleworkers to fill in what their main feeling was related to noise and added loss of concentration as an answer option. In three studies, an existing scale was used to measure concentration, namely the Checklist Individual Strength (CIS). These authors used a seven-point scale, ranging from low concentration to high concentration [7, 19, 31].

#### 3.4.5 Sleep quality

Some authors asked respondents to indicate how much trouble they experienced sleeping or how much their sleep was disturbed while WFH [6, 24, 29, 34]. Wütschert et al. [13] used an existing single-item scale for the evaluation of sleep-related problems that employees experienced during the past two weeks. Similarly, Bergefurt et al. [31] and Boegheim et al. [19] used the Single-item Sleep Quality Scale (PSQ). Other scales are the Athens Insomnia Scale (AIS) [32] and the Health at Work Survey of WHO [7, 19]. He et al. [18] used several measures to get an indication of employees’ sleep quality. They used online sleep diaries, the Pittsburgh Sleep Quality Index (PSQI), the Insomnia Severity Index (ISI), and actigraphy for objective measures. PSQI consists of seven constructs, namely subjective sleep quality, sleep latency, sleep duration, habitual sleep efficiency, sleep disturbances, use of sleeping medication, and daytime dysfunction. ISI is a seven-item survey which can be used to measure sleep difficulties and severity of insomnia symptoms. Last, actigraphy can be used to objectively monitor employees’ sleep, including sleep quality, wake-time activity, and intensity of movements in free-living settings.

#### 3.4.6 Fatigue

Only five studies investigated employees’ feelings of fatigue. Margariti et al. [15] used diaries and interviews, in which employees indicated to feel low, worried, positive, or slept well. These statements were used to get insights in respondents’ perception of fatigue. In three studies [7, 19, 31], the Checklist Individual Strength (CIS) was used, which consists of twenty items related to both fatigue and concentration. Respondents were asked to rate on a seven-point scale whether they felt tired, physically exhausted, weak, rested, etc. Last, Awada et al. [24] asked respondents to indicate their physical health status compared to the situation before WFH, of which feelings of fatigue was one symptom.

#### 3.4.7 Mood

Some authors did not use existing mood-scales, but asked employees to indicate if they experienced mood swings [6, 24], if noise affected their mood [29], or to indicate their moodiness [34]. Margariti et al. [15] used the Circumplex Model of Affect, which consists of the dimensions valence (i.e., pleasant-unpleasant or positive-negative), arousal (i.e., activation-deactivation or energetic-non energetic) and stance (i.e., low tension-high tension). The Plutchik’s wheel of emotions can also be used to capture teleworkers’ self-assessed emotions and mood [16]. Last, mood can be measured by the UWIST Mood Adjective Checklist, which consists of adjectives that can be summarized in the dimensions tense arousal (i.e., calm, relaxed – tense, nervous) and hedonic tone (happy, satisfied – sad, low-spirited) [7, 19, 31].

#### 3.4.8 Depression

Xiao et al. [6] and Awada et al. [24] did not use an existing scale to measure depressive symptoms. The other authors did use such scales, including the Depression, Anxiety, and Stress Scale (DASS-21) and the Four-item Patient and Health Questionnaire for Depression and Anxiety (PHQ-4). DASS-21 consists of three constructs, of which depression is one. It is measured by seven items, namely dysphoria, hopelessness, devaluation of life, self-deprecation, lack of interest or involvement, anhedonia, and inertia [12]. PHQ-4 consists of two short scales to measure anxiety disorders (Generalized Anxiety Disorder Scale, GAD-2) and depression disorders (Patient Health Questionnaire, PHQ-2) [7, 16, 19, 31].

#### 3.4.9 Engagement

In six studies the mental health indicator engagement was measured. Xiao et al. [6] and Awada et al. [24] asked teleworkers to rate their social engagement [7, 31]. Furthermore, the Oldenburg Burnout Inventory (OLBI) was used in three papers [7, 19, 31]. This scale consists of sixteen items, eight to measure (dis)engagement and eight to measure burnout. Employees are asked to rate these items on a four-point scale, ranging from strongly agree to strongly disagree. Last, Mäkikangas et al. [20] used the short three-item version of the Utrecht Work Engagement Scale. This scale consists of the items ‘I have felt bursting with energy while working’, ‘I have been enthusiastic about my work’, and ‘I have been immersed in my work’, which were all scored on a five-point scale.

#### 3.4.10 Burnout

In three studies, the Oldenburg Burnout Inventory (OLBI) was used to measure burnout with eight items that employees had to rate on a four-point scale [7, 19, 31]. In the fourth study, teleworkers were asked to indicate which disorders they suffered from during the COVID-19 lockdown, including eye disorders, headaches, concentration issues, and exhaustion [35]. Exhaustion has been scaled under burnout, because some authors (e.g., [37]) argued that burnout consists of emotional exhaustion, depersonalization, and lack of personal accomplishment.

### 3.5 Direction of relationships between physical home-workspace characteristics and mental health indicators

#### 3.5.1 Noise, acoustics, and privacy

Table 5 shows the direction of significant relationships between all physical workspace characteristics and mental health. An upward arrow indicates a positive relationship, a downward arrow a negative relationship, and a cross indicates that the direction of the relationship has not been defined in the study.

**Table 5:** Direction of relationships between physical workspace characteristics and mental health

Physical workspace characteristics	Mental health characteristics																			
	Concentration		Sleep quality		Mood		Stress		Productivity		Depression		Well-being		Fatigue		Engagement		Burnout	
Noise, acoustics, and privacy
Noise disturbance/ annoyance	↓	[14, 27, 31]	↓	[29]	↓	[29, 31]	↑	[27, 31]	↓	[31]
Uncomfortable noise													↓	[31]
Satisfaction with noise level	↑	[6, 19, 24]	↑	[6, 24]	↑	[6, 15, 19, 24]	↓	[6, 24]	↑	[5, 25, 32]	↓	[6]	↑	[19]	↑	[15,24]	↑	[6, 19, 24]
Comfortable acoustic environment													↑	[21, 22]
Sounds from others	X	[32]
Low privacy			↓	[13]
Acoustic quality									↑	[26]
Sound pressure level above 58dB					↓	[19]
Layout and design
Dedicated workroom	↑	[6, 31]	↑	[6]	↑	[6]	↓	[6]	↑	[5, 31]	↓	[6]	↑	[31]			↑	[6]
Variety of workspaces									↓	[5]
(Satisfaction with) spatial dimensions/ room size	X	[14]					↓	[23]
Single-family home	↑	[35]
Functionality of home-workspace																	↑	[20]
Satisfaction with layout									↑	[25]
Living space > 90m2																			↓	[35]
Light and daylight
Satisfaction with natural light	↑	[7]					↓	[7, 23]	↓	[24]
Satisfaction with artificial light					↑	[7]
Low level light exposure time			↓	[18]
High levels of sunlight	↑	[34]	↑	[34]	↑	[34]
Satisfaction with natural light, artificial light, glare	↑	[6]	↑	[6]	↑	[6, 15]	↓	[6]	↑	[25, 32]	↓	[6]					↑	[6]
Illuminance above 575 lux	↑	[19]															↑	[19]
Thermal comfort and temperature
Satisfaction with thermal environment	↑	[6, 32]	↑	[6]	↑	[6, 15]	↓	[6]	↑	[5, 25, 33]	↓	[6]					↑	[6]
Thermal comfort									↑	[26]
Biophilia and views
Access to garden/ private green	↑	[34]	↑	[34]	↑	[34]	↓	[12]			↓	[12]
Satisfaction with views outside	↑	[7]							↑	[25]
Satisfaction with greenery													↑	[7]
Absence of terrace/ patio
Access to outdoor space											↓	[16]
Natural elements in view outside	↑	[34]	↑	[34]	↑	[16, 34]
Potted plants			↑	[34]	↑	[34]
Indoor air quality and ventilation
Satisfaction with air quality	↑	[6, 14]	↑	[6, 24]	↑	[6]	↓	[6, 24]	↑	[25]	↓	[6]					↑	[6]
Fresh air quality					↑	[15]			↑	[26]
Look and feel
Blue/green wall colours					↑	[31]	↑	[31]
Change in wall colours									↑	[30]

Noise at home can be divided in indoor (e.g., systems and service, neighbours, family members, roommates) and outdoor noise sources (e.g., construction, traffic). Both indoor and outdoor noise disturbed employees’ sleep and negatively affected their mood. Especially noise by teleworkers’ children, partner or roommates made it difficult for them to concentrate on their job [29]. These sounds also had a negative effect on people’s well-being [22]. But noise from outdoors was also frequently mentioned to cause fatigue [15] and disturb teleworkers’ sleep [29]. Overall, uncomfortable noise levels distracted employees from their job, and reduced their well-being [31], and concentration levels [27]. The comfort of the acoustic environment was found to be important for employees’ psychological well-being [21] and concentration [32]. Torresin et al. [21] argued that the acoustic comfort could be improved by closing windows and doors, listening to music, and wearing noise-cancelling headphones.

Another predictor of mental health at home is satisfaction with noise. Those who were satisfied with noise felt less fatigued, depressed, stressed, indicated to have fewer mood swings and concentration difficulties [24] and felt more productive [25]. Boegheim et al. [19] also found that satisfaction with the noise level increased employees’ well-being and engagement, and diminished feelings of tension. During the COVID-19 pandemic, employees were less likely to report new mental health issues, such as depression, trouble sleeping, mood swings, decreased social engagement, and trouble concentrating, if they were satisfied with noise at home [6]. However, those who were dissatisfied with the privacy at home indicated more sleep problems [13]. Especially in shared rooms, noise annoyance was found to be higher, which could reduce teleworkers’ productivity, well-being, and concentration [21, 27].

#### 3.5.2 Light and daylight

Teleworkers’ satisfaction with the visual environment (i.e., natural light, electric light, and glare) influences their mental health. As Xiao et al. [6] argued, those who were satisfied with the visual environment had a smaller chance of reporting new mental health issues during the COVID-19 pandemic, including depression, trouble sleeping, mood swings, decreased social engagement, and trouble concentrating. Higher satisfaction with the visual environment also increases teleworkers’ productivity [25]. However, low levels of light exposure were found to be related to the misperception of sleep onset latency (i.e., discrepancy between subjective and objective sleep measures). This shows that low light exposure levels increase the chance of insomnia [18], while illuminance levels above 575lux lead to higher engagement [19].

Both access to daylight and artificial light are thus important for employees’ mental health. While satisfaction with artificial light significantly improves employees’ mood [7, 15], satisfaction with daylight reduces stress [7] and depressive symptoms [13]. Some teleworkers indicated that the daylight entrance at their home-workspace was suboptimal. Some of them relocated their desk to improve ambient conditions, while others used daylight-lamps to improve their mood and productivity [15]. Higher levels of sunlight, measured by the brightness level, were associated with lower self-reported moodiness, concentration, and sleep disturbance [34].

#### 3.5.3 Layout and design

Due to the COVID-19 pandemic, employees were obliged to WFH, even if their houses were not suitable for teleworking [5]. As Xiao et al. [6] indicated, those who did not have a dedicated workroom at home had a larger chance of reporting new mental health issues, including depression, trouble sleeping, mood swings, decreased social engagement, and trouble concentrating. In shared rooms, employees might have been more annoyed by noise, which reduced their well-being and productivity [27]. Some employees indicated to work in a variety of rooms at home, which might have reduced their productivity even further [5].

Furthermore, the spatial dimensions of the home were found to be important for teleworkers’ mental health. For instance, Fornara et al. [23] showed that satisfaction with the spatial dimensions of the home (i.e., in general, square footage, privacy, and natural light) reduced psychological distress. The spatial environment was also found to be important for employees’ concentration level [32]. Similarly, Muñoz-González et al. [35] argued that those who lived in single-family dwellings were less likely to experience concentration difficulties than those who lived in multi-family homes. They also showed that having access to a large living space (larger than 90m^2^) reduced the chance of mental problems, such as concentration issues and exhaustion. Mäkikangas et al. [20] included several home-office features to measure the home-office’s functionality, such as the adequacy of space and equipment to WFH, and the possibility to maintain a healthy work-life balance. They found that higher functionality of the home-work environment increased teleworkers’ work engagement significantly.

#### 3.5.4 Thermal comfort and temperature

One of the advantages of teleworking is having control over the indoor temperature at home, which might enhance teleworkers’ concentration level [14]. As Awada et al. [24] found, those who were dissatisfied with the temperature at home had trouble concentrating. Furthermore, thermal satisfaction (i.e., indoor temperature and humidity) [5, 33] and thermal comfort [26] were important for teleworkers’ productivity. Especially during the COVID-19 pandemic, satisfaction with the thermal environment might have prevented employees from developing new mental health issues, such as depression, trouble sleeping, mood swings, decreased social engagement, and trouble concentrating [6].

#### 3.5.5 Biophilia and views

In general, satisfaction with greenery predicted higher well-being [7]. Having access to greenery (e.g., a garden or patio) reduced teleworkers’ feelings of stress [12] and depressive symptoms [16]. However, those who did not have access to a patio or terrace suffered from a lack of concentration [35]. Spano et al. [34] argued that the presence of potted plants within the home-workspace was associated with lower self-reported moodiness and sleep disturbance.

Next to the presence of greenery indoors or outdoors, several researchers studied the influence of views outside on mental health while WFH. Generally, satisfaction with views outside was found to be related to higher concentration [7]. More specifically, having natural views outside was associated with fewer depressive symptoms, a more positive mood [16], and lower self-reported moodiness, concentration, and sleep disturbance [34].

#### 3.5.6 Indoor air quality and ventilation

Teleworkers who were satisfied with the air quality at home had less trouble sleeping and experienced less mental stress [15, 24]. They also experienced a more positive mood [15], rated their productivity to be higher [26], and indicated enhanced concentration due to better air quality [14]. As Xiao et al. [6] indicated, during the COVID-19 pandemic, the chance of teleworkers reporting new mental health issues, such as depression, trouble sleeping, mood swings, decreased social engagement, and trouble concentrating, was lower when they were satisfied with the air quality at home.

#### 3.5.7 Look and feel

Blue or green wall colours both have a positive effect on stress and mood, which indicates that employees felt more stressed, but also felt happier and more satisfied when their wall colours at home were blue or green [31]. Furthermore, those who changed the wall colours at home rated their productivity higher. The use of wall colours might positively affect teleworkers’ mood, which could, in turn, improve their productivity [30]. Other relationships between look and feel and mental health have not been found in the included papers.

## 4 Discussion

This study aimed to systematically review existing research on the relationship between physical home-workspace characteristics and mental health. It provided an overview of existing knowledge for more evidence-based design of the home workspace and identified research gaps for future research. Another scientific contribution is that this study forms a holistic basis for future research on employees’ mental health while WFH, by summarizing a list of potential measures. This study showed that home-work environmental research is a multi-disciplinary research topic, shown by the four distinguished research fields (i.e., medicine and health, occupation and ergonomics, building science, and psychology). As Appel-Meulenbroek et al. [38] indicated, the workplace research field in general benefits from multi-disciplinarity, because researchers’ expertise in research methods- and -findings can be combined to fill existing research gaps.

A practical implication is that this study provided insights in how the home-work environment should be designed to optimize mental health and reduce mental issues. Most papers on this relationship were written during the COVID-19 pandemic, while only one study [14] was written before the pandemic. This shows that the interest in the relationship between the home-work environment and mental health has rapidly grown due to the pandemic. In this period, WFH was obliged for many employees. Because some employees might decide to (partly) remain WFH after the pandemic, workplace managers and employers should consider how they can support the optimization of the physical characteristics of the home-work environment as well.

Out of the 27 included studies, 23 used a cross-sectional approach, meaning that observations were measured at only one point in time. In the beginning of the pandemic, WFH might have been observed as a temporary situation and longitudinal research might have been considered too burdensome. Another reason for the few longitudinal studies might be a lack of data in the pre-pandemic situation (e.g., because employees were working at the office before the pandemic) [22]. Furthermore, several studies used snowball sampling techniques, which might have caused selection bias. As a result, the quality of these studies was assessed as low (either 0% or 25%). Especially for physical home-workplace characteristics that have been studied less frequently, such as biophilia and views outside, and look, feel and colour, the lower study quality might be critical. Therefore, in future studies, especially a longitudinal approach should be considered, because it allows the observation of changes in behaviour and experience of teleworkers over a longer period [7]. As some mental health indicators are a result of prolonged demands and/or lack of adequate resources, it is valuable to measure the effect of workspaces on the longer term.

In addition, it was found that only a few studies used objective measures for the physical workspace characteristics at home. For example, He et al. [18] measured light exposure levels by using actigraphy wristwatches. Humidity loggers that were placed at individuals’ desks have been used to measure air temperature, relative humidity [32, 33], CO_2_ concentration, and PM_2.5_ mass concentration levels [32]. In the office-environment, the use of environmental sensors and physiological measures to accurately assess human-building interactions has become more advanced. These ‘living lab’-type investigations allow the control and continuous monitoring of changes in employees’ behaviour and health [39]. The living lab approach has not yet been applied to the home-work environment. However, the use of actigraphy wristwatches or humidity loggers at individuals’ desks allow to objectively measure IEQ-related aspects of the residential environment.

This study also shows that noise, acoustics, and privacy were most frequently studied, especially in relation to productivity and stress. As Bergefurt et al. [31] showed, noise is the largest distractor in the home-work environment. It is therefore not surprising that most reviewed papers focussed on the influence of noise, acoustics, and privacy on employees’ mental health. Noise nuisance mainly depends on the size and composition of both the family and the home. During the COVID-19 pandemic, the absence of a dedicated workroom and having a larger family were found to be distracting [31]. Under these circumstances, employees might have been more annoyed by noise. The noise sources at home are also different from those at the office (e.g., noise from family members or neighbourhood noise) [4], which might explain researchers’ focus on noise in the home-work environment.

Current results also showed that the reviewed papers focussed mostly on productivity, concentration, and sleep quality, followed by the more emotion-focussed indicators mood and stress. For the office environment, studies mainly focussed on productivity [8], while for the home-work environment, several other mental health indicators were also frequently examined. At the office, employees might experience a sense of social control, more psychological demands, or lower decision authority [40]. They might therefore be more focussed on reaching their goals and targets in the presence of their colleagues. On the other hand, the home is a place for relaxation and entertainment [4]. It is challenging to keep a clear boundary between work and non-work at home [41], which might explain the focus on the emotion-related indicators of mental health.

### 4.1 Strengths and limitations

The use of the PRISMA method allowed for systematically reviewing literature on the relationship between physical workspace characteristics and mental health indicators in the home-work environment. Although inter-study evaluations were not performed due to substantial differences in research designs between included studies, the review does show which relationships have frequently been studied and the direction of these relationships. However, some limitations remain. First, the eligibility criteria might have limited the number of papers that were found. Only empirical studies, including longitudinal, prospective, and cross-sectional research designs, were considered here, while grey literature, proceedings, theoretical papers, and reviews were left out. Another limitation is that only studies that were written in English were included in this review. Furthermore, the quality assessment was based on information that was included in the papers, which was subjectively assessed by the first author. Although the MMAT quality assessment format was used, the scoring process was still subjective. Nonetheless, it has provided valuable insights for academics and practitioners to further study and optimise the home workspace.

## 5 Conclusion

This systematic scoping review has listed current empirical evidence on the relationship between physical home-workspace characteristics and mental health. It showed that 26 of the 27 papers were written during the COVID-19 pandemic, which indicates the increased interest in the potential influence of the home-work environment on employees’ mental health. However, almost all these studies used a cross-sectional approach, leaving objective measures of IEQ-related aspects undetermined. Future research could use a living-lab approach to determine these objective measures at home. Both researchers and practitioners can use existing and future knowledge to support a healthy home-work environment more effectively.

## Ethical approval

This study, as a literature review, is exempt from Institutional Review Board approval.

## Conflict of interest

The authors declare that they have no conflict of interest.
